# Evaluating the Variation of Intraocular Pressure With Positional Change During Colorectal Laparoscopic Surgery: Observational Study

**DOI:** 10.2196/11221

**Published:** 2018-09-04

**Authors:** Parveen Vitish-Sharma, Anthony J King, Richard Stead, John Sharp, Ali Abbas, Boliang Guo, Christopher Gornall, Charles Maxwell-Armstrong, Austin G Acheson

**Affiliations:** ^1^ Nottingham University Hospital NHS Trust Nottingham United Kingdom; ^2^ Institute of Mental Health University of Nottingham Nottingham United Kingdom

**Keywords:** laparoscopic, colorectal, intraocular pressure, Trendelenburg

## Abstract

**Background:**

The incidence of perioperative visual loss following colorectal surgery in the US is quoted as 1.24 per 10,000. Raised intraocular pressure (IOP) during extreme Trendelenburg position leading to reduced optic nerve perfusion is thought to be a cause.

**Objective:**

To assess the effect of the degree of Trendelenburg tilt and time spent in Trendelenburg on IOP during laparoscopic colorectal surgery.

**Methods:**

Fifty patients undergoing laparoscopic colorectal surgery were recruited. A Tonopen XL applanation tonometer was used to take IOP measurements hourly during surgery, and each time the operating table was tilted. A correlation coefficient for the degree of Trendelenburg tilt and IOP was calculated for each patient. Group 1 included patients undergoing a right-sided colonic procedure, and Group 2 included all left-sided colonic operations.

**Results:**

The mean age of Group 1 participants (n=25) was 69 years (SD 14), and Group 2 (n=25) was 63 years (SD 16; *P*>.05). The average length of surgery for Group 1 was 142 minutes (SD 48), and Group 2 was 268 minutes (SD 99; *P*≤.05). The mean maximum degree of Trendelenburg tilt in Group 1 was 10 (SD 7) and Group 2 was 19 (SD 6; *P*≤.05). The mean IOP increase was 9 mm Hg (SD 5) for Group 1 and 15 mm Hg (SD 5) in Group 2 (P≤.05). An overall correlation coefficient for the degree of Trendelenburg tilt and IOP change (n=48) was .78.

**Conclusions:**

There is a strong correlation between IOP elevation during laparoscopic colorectal surgery and the degree of Trendelenburg tilt. This may be significant for patients undergoing prolonged surgery and especially those with glaucoma.

## Introduction

### Background

Trendelenburg positioning is commonly used during laparoscopic colorectal surgery to allow the use of gravity to move the small bowel out of the pelvis and provide the surgeon with adequate views. The degree of tilt and time spent in these positions varies depending on the type of resection, the complexity of the case, and the surgeon. Trendelenburg positioning and pneumoperitoneum used during laparoscopic surgery lead to an increase in central venous pressure (CVP). An increase in CVP leads to a rise in episcleral venous pressure which in turn increases intraocular pressure (IOP). As the eye has limited distensibility, it only requires a small change in aqueous humor volume for IOP to change significantly. Problems in venous drainage can lead to a reduced arterial blood supply, reducing oxygen delivery to the optic nerve, and result in ischemia and neovascularization [1].

Postoperative vision loss (POVL) is a serious complication which significantly affects the quality of life. The incidence of POVL has gradually increased. The cause is thought to be multifactorial: (1) an increase in more complex surgeries being performed, and (2) patients with multiple comorbidities who are at higher risk of postoperative complications [2,3]. In cases where the cause is not identified (eg, foreign body in the eye), the most common explanation is optic nerve ischemia [2,4]. Ischemia may be the result of anemia, hypotension, blood loss, or raised IOP leading to optic nerve [2,5,6].

### Objective

This study aimed to look at how the degree of Trendelenburg tilt during laparoscopic colorectal surgery affects IOP.

## Methods

### Study Design

This study was a clinically based prospective observational trial. The study was reviewed and approved by the Northampton Research Ethics Committee (protocol number: 11GA019, April 2012) and undertaken as per the tenets of the Council of Helsinki. All patients undergoing planned laparoscopic colorectal resection under the colorectal surgery service at Nottingham University Hospital were invited to participate in the study. Patients undergoing a right-sided colonic procedure were included in Group 1, and those undergoing left-sided colonic procedures (including subtotal resections) were included in Group 2. Participants were divided into these groups because left-sided colon procedures were hypothesized to spend longer in a steeper Trendelenburg tilt compared to those only undergoing right-sided procedures. Patients with a history of significant ocular disease other than glaucoma, or an allergy to latex were excluded from this study. Patients expressing an interest in participating were given an information leaflet and those who were willing to join signed a consent form before study intervention.

Demographic data (1) gender, (2) age, (3) smoking history, (4) comorbidities, and (5) medication history was collected from each patient. Baseline eye examinations were also performed including: (1) best corrected visual acuity, (2) gonioscopy, (3) central corneal thickness, (4) Goldmann applanation tonometry, (5) and Tonopen XL applanation tonometer. The Tonopen XL applanation tonometer measurements were repeated after lying the patient supine for 5 minutes. They were collected after administering 1% tetracaine eye drops and repeated to obtain an average of 3 readings at 5% accuracy.

On the day of surgery, baseline IOP was taken in the right eye using the Tonopen XL applanation tonometer. IOP measurements were repeated in the right eye at the following points during surgery: (1) after induction of general anaesthetic, (2) at the start of surgery, (3) 5 minutes after pneumoperitoneum was created, (4) every hour after the start of surgery, (5) 5 minutes after the table was tilted at any point during surgery, and (6) at the end of surgery. The timing of these readings was documented along with the angle of the table tilt, positive end expiration pressure (PEEP), expired carbon dioxide (CO_2_) level, mean arterial pressure (MAP), and pulse rate.

Spinal or thoracic anesthesia was administered at the start of each operation. Spinal anesthesia consisted of up to 500 µg of intrathecal diamorphine and up to 20 mg of bupivacaine. Epidural anesthesia was maintained with 0.125% levobupivacaine and 4 µg/mL of fentanyl. Induction of general anesthesia included 25-50 µg/kg of midazolam, remifentanil (0.5-1 µg/kg) or fentanyl (1-2 µg/kg), propofol (1-2.5 mg/kg), and neuromuscular blockade with either rocuronium or atracurium was given. Anesthesia was maintained with intraoperative target-controlled remifentanil infusion at (0.05-2.0 µg/kg per minute) in addition to oxygen, air, and desflurane.

### Statistical Analysis

Data were analyzed with an unpaired *t* test for comparison of IOP change, length of surgery, and the degree of tilt used between the 2 groups. A paired *t* test was used for comparison of IOP before and after the induction of a pneumoperitoneum and maximum IOP increase with the maximum degree of Trendelenburg tilt during surgery. A Pearson’s correlation coefficient between the degree of tilt measurements and IOP was calculated for each patient. The individual correlation coefficients were then pooled using a meta-analytic approach considering the different number of readings and potential heterogeneity between patients. The Stata code metan was used to perform meta-analysis modeling. All correlation coefficients were normally distributed into Fisher Z, and the pooled Fisher *z* scores (95% CI) were then transformed back to a correlation coefficient with 95% CI using the z to r transformation equation.

**Figure 1:**
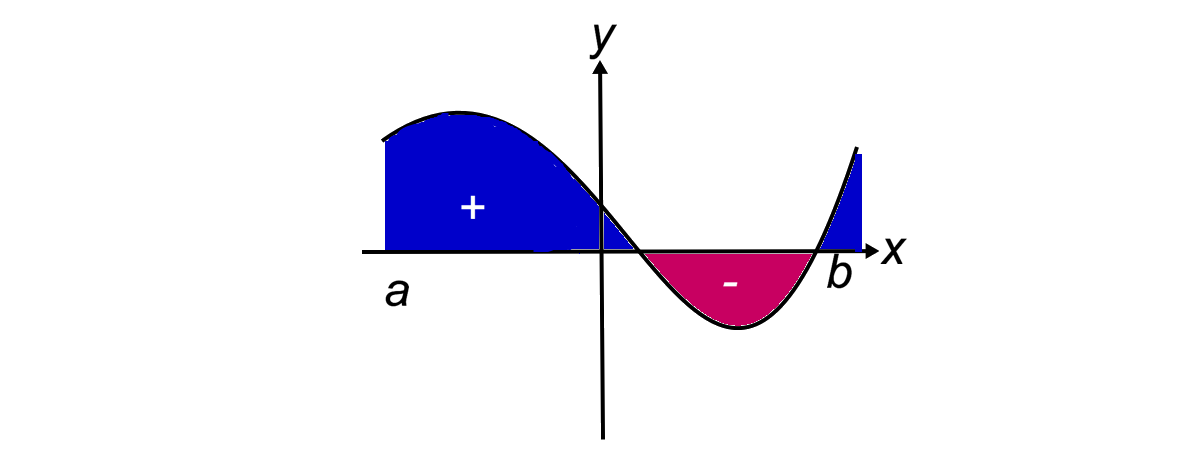
Tilt AUC calculation method= start of surgery, b= end of surgery, y-axis= degree of head down tilt, x-axis= time in minutes. For ‘change in IOP’ AUC: y-axis= change in IOP in mmHg, x-axis = time in minutes.

Further analysis to incorporate the length of time spent at various degrees of Trendelenburg tilt was carried out by determining the area under the curve (AUC). This was calculated by plotting the time from the start of surgery against the degree of table tilt in either an upward (negative) or downward (positive) position. The Trendelenburg tilt was recorded as positive and reverse Trendelenburg as negative. The AUC was determined by multiplying the degree of table tilt by the time spent in that position in minutes. If the table was in the upward head position, this value would be negative (ie, the negative y-axis portion of graph Figure 1). All these were added up to give a cumulative tilt AUC by the product of degrees and minutes. For example, time spent in the upward head position gave a negative value which was effectively subtracted from the total AUC when added to the portion of the curve where the patient was in the Trendelenburg position. The change in IOP AUC was calculated similarly, with IOP change from baseline plotted on the y-axis, and the x-axis time in minutes from the start of surgery. The change in IOP was calculated by subtracting the baseline IOP from the IOP measurements taken during surgery. If the IOP went below the baseline IOP, this was plotted on the negative y-axis which effectively subtracted from the overall cumulative change in IOP AUC.

A multilevel mixed analysis was carried out comparing the following variables to the change in IOP AUC that occurred at each time point in each patient. The variables analyzed included: (1) time from the start of surgery (minutes), (2) AUC, (3) pneumoperitoneum pressure in millimeters of mercury (mm Hg), PEEP, (4) CO_2_, and (5) MAP.

## Results

Fifty-five patients were enrolled in this study of which 5 withdrew their consent on the day of surgery. Group 1 and Group 2 each consisted of 25 patients. Twenty-six (52%) were male, and 24 (48%) were female with a mean of 66 (SD 16) years of age. Three (6%) of the patients were graded as American Society of Anesthesiologists (ASA) 1, 43 (86%) were ASA 2, and 4 (8%) were ASA 3. The mean body mass index (BMI) was 27 (SD 5) kilograms per meter squared (kg/m^2^). Table 1 summarizes the demographics of patients in each group. Table 2 details the operative procedures performed in each group.

### Correlation Between Tilt Area Under the Curve and Intraocular Pressure

**Figure 2:**
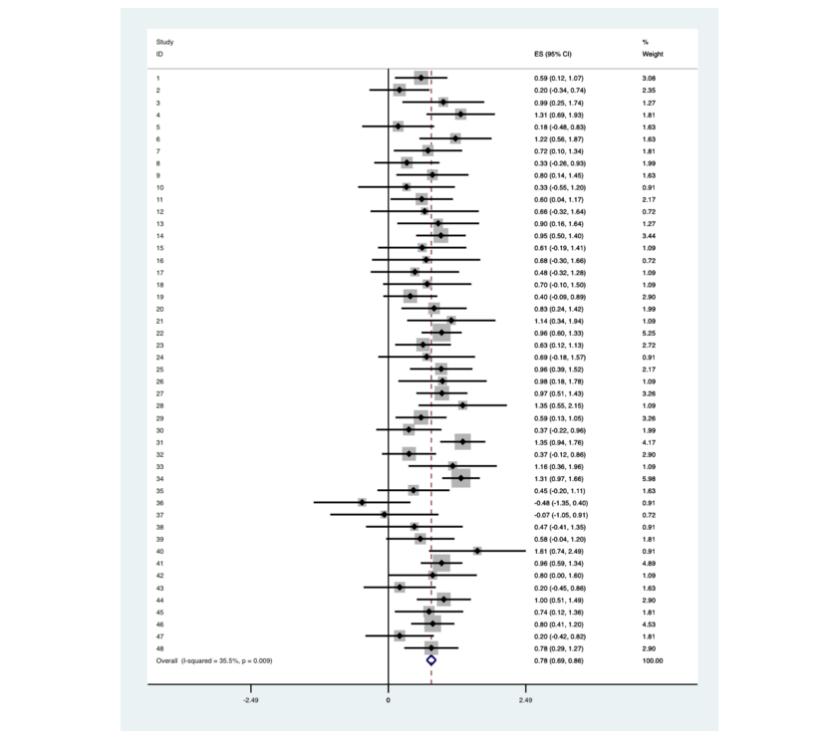
A graph of the intra-operative data collected for patient 1. It shows a strong correlation between the degree of tilt and the IOP measurements taken using the Tono-Pen XL.

Correlation between the degree of tilt AUC and IOP was analyzed for each patient. Meta-analysis of Pearson’s correlation coefficient between the degree of tilt and IOP was estimated at *r*=.78 with heterogeneity chi-squared (X^2^_47_=72.9, *P*=.009) indicating there is a significant positive correlation between the IOP and the degree of Trendelenburg tilt (Figure 2). Two patients were excluded from this analysis as they remained supine (at degree zero) which did not allow for a Pearson’s correlation calculation.

### Comparison of Tilt and Intraocular Pressure Between Groups 1 and 2

Comparison between Group 1 and Group 2 was performed using an unpaired *t* test for maximum IOP increase and maximum Trendelenburg tilt. There was a significant difference between the 2 groups. For the maximum IOP increase from baseline, the *t* score was 3.89 with 95% CI –8.79 to –2.81, *P*<.001. For the maximum Trendelenburg tilt, the *t* score was 4.72 with 95% CI –12.64 to –5.09, *P*<.001 (Table 3).

### Multilevel Analysis of Factors That May Affect Intraocular Pressure

A multilevel mixed analysis compared the change in IOP AUC at each time point with each variable measured to include: (1) time from start of surgery, (2) tilt AUC, (3) pneumopressure, (4) PEEP, (5) CO_2_ level, and (6) MAP. For this analysis, all patients were included. The output from this analysis is detailed in Table 4.

The statistical analysis carried out showed that the critical factors affecting IOP rise was the length of surgery, tilt AUC, and expired CO_2_.

### Analysis of the Effect of Pneumoperitoneum on Intraocular Pressure

The effect of pneumoperitoneum on IOP was assessed by comparing the IOP measured 5 minutes after the induction of pneumoperitoneum to the maximum IOP rise that occurred intra-operatively. The surgeon would create the pneumoperitoneum and carry out a diagnostic laparoscopy while supine before tilting the patient, and an IOP measurement would be taken. A two-tailed *t* test was carried out with *t*=7.79, 95% CI 6.20 to 9.38, *P* ≤.001 (Table 5). In 1 patient, we were unable to measure IOP immediately after the induction of pneumoperitoneum as a central line was being placed, they were therefore excluded from the *t* test analysis.

**Table 1:** Patient demographic data comparison between Group 1 and Group 2.

Parameter		Group 1	Group 2	
**Gender, n (%)**				
	Male	15 (30)		11 (22)
	Female	10 (20)		14 (28)
Age (years), mean (SD)		69 (14)	63 (16)	
BMI^a^ (kg/m^2^), mean (SD)		26 (4)	28 (6)	
**ASA^b^grade, n (%)**				
	I	1 (2)		2 (4)
	II	20 (40)		23 (46)
	III	4 (8)		0 (0)
**Operative time (minutes), n (%)**				
	<100	5 (10)		0 (0)
	100-199	18 (36)		4 (8)
	200-299	3 (6)		11 (22)
	300-399	0 (0)		3 (6)
	400-499	0 (0)		4 (8)
	>500	0 (0)		2 (4)
**Length of stay (days), n (%)**				
	<3	2 (4)		0 (0)
	3-5	12 (24)		12 (24)
	6-10	7 (14)		8 (16)
	>10	3 (6)		5 (10)
Deaths, n (%)		1 (2)^c^	0 (0)	
**Trendelenburg tilt (degree), n (%)**				
	<14	18 (36)		5 (10)
	14-20	7 (14)		12 (24)
	>20	0 (0)		8 (16)
**Blood loss (mL), n (%)**				
	<100	15 (30)		17 (34)
	100-500	7 (14)		3 (6)
	>500	3 (6)		5 (10)

^a^BMI: body mass index.

^b^ASA: American Society of Anesthesiologists.

^c^Day 2 from a chest infection.

**Table 2:** Operation details for all 50 patients.

Parameter		n (%)
**Group 1**		
	Laparoscopic right hemicolectomy	24 (96)
	Laparoscopic colotomy	1 (4)
**Group 2**		
	Laparoscopic anterior resection	15 (60)^a^
	Laparoscopic Hartmann’s	3 (12)^b^
	Laparoscopic subtotal colectomy	4 (16)^a^
	Laparoscopic panproctocolectomy	1 (4)
	Laparoscopic completion proctectomy and ileoanal pouch	1 (4)^a^
	Extralevator abdominoperineal resection	1 (4)

^a^One converted to open.

^b^Two converted to open.

**Table 3:** Overall mean length of surgery, baseline, and mean rise in intraoperative pressure between Group 1 and Group 2.

Parameter	Group 1	Group 2	*P* value
Age (years), mean (SD)	69 (14)	63 (16)	.15
Baseline IOP^a^ (mm Hg), mean (SD)	16 (4)	17 (2.9)	.64
Length of surgery (minutes), mean (SD)	142 (49)	268 (99)	<.001
Maximum Trendelenburg tilt (degree), mean (SD)	10 (5)	15 (5)	<.001
Maximum increase from baseline IOP intraoperatively (mm Hg), mean (SD)	9 (5)	15 (5)	<.001

^a^IOP: intraocular pressure.

**Table 4:** Regression analysis outcome for all 50 patients.

Change in intraocular pressure AUC^a^	Coefficient (SE)	*z* value	Coefficient (95% CI)	*P* value
Time from start of surgery	4.33 (0.49)	8.88	4.33 (3.37 to 5.28)	<.001
Tilt AUC	0.48 (0.04)	12.66	0.48 (0.41 to 0.56)	<.001
Pneumopressure	–2.46 (4.16)	–0.59	–2.46 (–10.62 to 5.69)	.55
PEEP^b^	25.05 (26.54)	0.94	25.05 (–26.97 to 77.07)	.35
CO_2_^c^	121.69 (47.06)	2.59	121.69 (29.45 to 213.93)	.01
MAP^d^	1.46 (1.84)	0.79	1.46 (–2.16 to 5.07)	.43

^a^AUC: area under the curve.

^b^PEEP: positive end-expiratory pressure.

^c^CO_2_: concentration of expired carbon dioxide.

^d^MAP: mean arterial pressure.

**Table 5:** Comparative data for maximum intraoperative pressure (IOP) increase during the operation and the increase following pneumoperitoneum induction.

Parameter	N	Mean (SD)
Maximum IOP increase	50	12 (6)
IOP rise following pneumoperitoneum	49	4 (6)

## Discussion

### Principal Findings

Laparoscopic surgery is the preferred approach for most colorectal resections. The advantages include (1) smaller incisions, (2) reduced blood loss, (3) less postoperative pain, and (4) reduced recovery time [7]. Trendelenburg positioning is used during laparoscopic colorectal surgery and other specialties including urology and gynecology to utilize gravity as a form of retraction. It allows the small bowel to fall out of the pelvis away from the operative field during left-sided resections. During a right hemicolectomy, the Trendelenburg position is used to help move the small bowel away during the cecal dissection. Our study found the degree of tilt used and the time spent in Trendelenburg is significantly lower in right-sided resections compared to left-sided resections. During a right hemicolectomy, we found that a reverse Trendelenburg position is often used when mobilizing the hepatic flexure. This was why we used tilt AUC as a measure for part of our analysis to consider the time spent in the reverse Trendelenburg as well as the Trendelenburg. As the reverse tilt was measured as a negative tilt, the amount of time spent in the reverse position reduced the overall AUC value. The maximum Trendelenburg tilt was significantly different between the 2 groups. For Group 1 the mean maximum Trendelenburg tilt was 9.70, and for Group 2 it was 15.10.

An IOP above 25 mm Hg is considered pathological [8]. Chauhan et al [9] looked at the effect of raised IOP in rats. Their data suggested changes were dependent on the peak increase in IOP. They found a peak increase of 15 mm Hg in IOP resulted in extensive axonal loss (mean loss of 69.2%), and a peak increase of 20 mm Hg in IOP resulted in profound axonal structural loss (mean reduction of 76.7%). They concluded that optic nerve axonal damage was related to the peak increase in IOP with a change of 10 mm Hg or more leading to damage of the optic nerve [9]. The length of time that IOP is raised has an additional cumulative effect [10,11]. Similar findings were also made by Morrison et al [12]. Our results showed a significant difference in the maximum change in IOP with a mean IOP rise of 9.3 mm Hg, versus 15.1 mm Hg in Group 1 and 2, respectively.

Grosso et al [10] compared 3 groups of patients: (1) those undergoing laparoscopic surgery supine, (2) laparoscopic surgery in Trendelenburg position, and (3) open surgery in a supine position. They looked at the effect of pneumoperitoneum (12-14 mm Hg) on IOP and found a mean rise of 4 (range 0-11.2) mm Hg, which was comparable to our 4.43 mm Hg rise, following pneumoperitoneum. The mean increase following 45 minutes after the start of surgery was 5.05 mm Hg in the Trendelenburg group versus 4.23 mm Hg in the laparoscopic group not placed in Trendelenburg [10]. In our study, we compared the IOP rise that was measured 5 minutes following induction of pneumoperitoneum to the overall maximum IOP rise that occurred during surgery. This gave a mean rise of 4.43 mm Hg following pneumoperitoneum induction compared to an overall rise of 12.22 mm Hg. This too was statistically significant suggesting the creation of pneumoperitoneum alone (at 11-14 mm Hg) that was used on our patients does not cause a clinically significant increase in IOP.

Awad et al [13] also looked at the effect of steep Trendelenburg positioning on IOP during robotic prostatectomy [13]. Their analysis revealed PEEP, duration of surgery, end-tidal CO_2_ levels, and MAP were all significant predictors of IOP change during surgery. The Grosso et al [10] and Awad et al [13] dataset varied from our study as their patients were placed in Trendelenburg position at the same degree of tilt and IOP measurements were taken at specific time points. In our study, the degree of Trendelenburg varied as did the time spent in Trendelenburg. This allowed us to assess the effect of time and position steepness on IOP. Our analysis also showed that the length of surgery, time spent in Trendelenburg position, the degree of Trendelenburg tilt, and expired CO_2_ levels were significant factors for change in IOP during surgery.

Glaucoma affects 2% of the population over the age of 40 years and this increases with age [14]. However, only half of those with glaucoma know they have it, meaning 50% do not know. Also, another 3%-5% of the population over this age suffer from ocular hypertension which is a risk factor for the development of glaucoma. Therefore, potentially 1 in 50 patients undergoing a laparoscopic colorectal resection could have glaucoma, but only 50% of these would have been diagnosed.

Our study also showed the degree of Trendelenburg tilt was strongly correlated with IOP, with a coefficient value of .78 (*P*=.009). During the study, we also observed that by reducing the tilt even by a few degrees, the IOP would reduce almost immediately, which is of great clinical significance. This may be a useful mechanism to prevent sustained IOP elevation during surgery when prolonged surgery is being undertaken. It may also benefit patients in whom there are concerns that optic nerve ischemia of prolonged IOP elevation may be risky such as patients known to have glaucoma.

### Limitations

There were limitations to our study, including the use of Tonopen instead of the gold standard Goldmann applanation tonometer for IOP measurement [15]. Studies have shown that taking an average of 2 readings, the accuracy of the Tonopen is significantly increased. In our study, we took 3 measurements and used the average. Other studies have shown this improves the accuracy of Tonopen measurements and are similar to the Goldmann applanation tonometer [15-17]. Measuring IOP in only 1 eye can also be a potential limitation. However, Grosso et al [10] measured IOP in both eyes at each time point and found minimal difference between the left and right eye [10,18]. A further limitation to our study was due to the IOP measurements being available throughout surgery to the anesthetist and surgeon. If the IOP measurements were high (>30 mm Hg), it prompted the anesthetist to ask for a reduction in the Trendelenburg tilt. In the few patients in which this was done, this led to the observation that reducing the Trendelenburg tilt by only 2 degrees, there was a decrease in IOP. Also, by returning the patient to supine, the IOP returned to near baseline after only 5 minutes of moving the table.

Vision loss is a significant potential complication of steep Trendelenburg positioning. However, even where catastrophic vision loss does not occur, sustained IOP elevation may result in some subclinical optic nerve damage. This may increase the risk of later vision loss [9], particularly in those patients who have preexisting optic nerve damage or develop optic nerve pathology later in life.

### Conclusion

In conclusion, our results showed a strong correlation between the degree, duration of the Trendelenburg tilt, and IOP during laparoscopic colorectal surgery. Significant and prolonged IOP elevation occurred in a proportion of patients which is of clinical significance when operating on patients with glaucoma. By reducing the degree of Trendelenburg tilt during laparoscopic colorectal surgery, the IOP rise can be reduced. Additional studies to consider intraoperative breaks from Trendelenburg or IOP screening preoperatively with targeted therapy to prophylactically reduce IOP in patients undergoing a lengthy surgical procedure requiring Trendelenburg positioning would be of clinical value.

## Abbreviations

AUC: area under the curve
BMI: body mass index
CO2: carbon dioxide
CVP: central venous pressure
IOP: intraocular pressure
MAP: mean arterial pressure
PEEP: positive end-expiratory pressure
POVL: postoperative vision loss

## References

[1] Craven ER: Raised Episcleral Venous Pressure (2004). Ophthalmology.

[2] Frost EA: Visual loss after anesthesia different causes: different solutions-a review (2010). Visual loss after anesthesia different causes: different solutions--a review. Middle East J Anaesthesiol.

[3] Warner MPVL (2006). Postoperative visual loss: experts, data, and practice. Anesthesiology.

[4] Molloy BL (2011). Implications for postoperative visual loss: steep trendelenburg position and effects on intraocular pressure. AANA J.

[5] Dunker S, Hsu HY, Sebag J, Sadun AA (2002). Perioperative risk factors for posterior ischemic optic neuropathy. J Am Coll Surg.

[6] Pinkney T, King A, Walter C, Wilson T, Maxwell-Armstrong C, Acheson A G (2012). Raised intraocular pressure (IOP) and perioperative visual loss in laparoscopic colorectal surgery: a catastrophe waiting to happen? A systematic review of evidence from other surgical specialities. Tech Coloproctol.

[7] COLOR Study Group (2000). COLOR: a randomized clinical trial comparing laparoscopic and open resection for colon cancer. Dig Surg.

[8] Cunningham AJ, Barry P (1986). Intraocular pressure--physiology and implications for anaesthetic management. Can Anaesth Soc J.

[9] Chauhan B, Pan J, Archibald M, LeVatte T, Kelly M, Tremblay François (2002). Effect of intraocular pressure on optic disc topography, electroretinography, and axonal loss in a chronic pressure-induced rat model of optic nerve damage. Invest Ophthalmol Vis Sci.

[10] Grosso A, Scozzari G, Bert F, Mabilia MA, Siliquini R, Morino M (2013). Intraocular pressure variation during colorectal laparoscopic surgery: standard pneumoperitoneum leads to reversible elevation in intraocular pressure. Surg Endosc.

[11] Sultan M, Mansberger S, Lee Paul P (2009). Understanding the importance of IOP variables in glaucoma: a systematic review. Surv Ophthalmol.

[12] Morrison J, Moore C, Deppmeier L, Gold B, Meshul C, Johnson E C (1997). A rat model of chronic pressure-induced optic nerve damage. Exp Eye Res.

[13] Awad H, Santilli S, Ohr M, Roth A, Yan W, Fernandez S, Roth S, Patel V (2009). The effects of steep trendelenburg positioning on intraocular pressure during robotic radical prostatectomy. Anesth Analg.

[14] National Institute for Health and Care Excellence.

[15] Okafor KC, Brandt JD (2015). Measuring intraocular pressure. Curr Opin Ophthalmol.

[16] De Smedt S: Noninvasive intraocular pressure monitoring: current insights (2015). Noninvasive intraocular pressure monitoring: current insights. Clin Ophthalmol.

[17] Lasseck J, Jehle T, Feltgen N, Lagrèze WA (2008). Comparison of intraocular tonometry using three different non-invasive tonometers in children. Graefes Arch Clin Exp Ophthalmol.

[18] Sit A, Liu J, Weinreb Robert N (2006). Asymmetry of right versus left intraocular pressures over 24 hours in glaucoma patients. Ophthalmology.

